# Department of Surgery

**Published:** 1900-07

**Authors:** W. E. A. Wyman, J. A. Sloan, Robert Jay

**Affiliations:** Burlington, Wis.; San Francisco, Cal.; West Liberty, Ia.


### DEPARTMENT OF SURGERY.

By W. E. A. Wyman, M.D.V., V.S.,
BURLINGTON, WIS.

J. A. Sloan, B.Sc., M.D.V.,
SAN FRANCISCO, CAL.

Dr. Robert Jay, M.D.V.,
WEST LIBERTY, IA.

---

### Penis and Sheath.

There are two diseases of the free portion of the penis and its cutaneous fold, the prepuce or sheath, which are very frequently met with, viz.: acrobustitis and balanitis.

*Acrobustitis*, inflammation of the sheath, or more particularly its mucous-membrane-like lining,

*Balanitis*, inflammation of the pendulous portion of the organ of copulation.

The former, at times quite stubborn to treat, and thus annoying, is primarily a sequel of emasculation, and therefore more commonly seen in the gelding. Inactivity atrophy of the penis is the natural consequence of castration. The penis becomes more deeply retracted into its protective fold, the sheath, and often remains within the preputial cavity during micturition. Accumulation and decomposition of the urine result in irritation and inflammation of the lining membrane of the sheath; eventually the glans becomes involved in the inflammatory process, and the above-referred-to state, balano-acrobustitis, exists.

Nevertheless, it occurs also in the stallion, where concretions and decomposing preputial smegma, the product of the numerous sebaceous preputial glands, of peculiar smell and dark gray in color, may represent the offending agent.

Acrobustitis following the "cleaning of the sheath," especially by attendants with long finger-nails or very broad hands, as also by the introduction of pepper, etc., during colics or imaginary renal troubles, is common.

While an acrobustitis in the gelding usually precedes balanitis, the reverse holds true in the stallion. With him balanitis follows the act of copulation, as through an improperly directed penis, undue friction in the vagina, its entrance into the rectum, etc. Finally, neoplasms may also give rise to this condition.

The first indication of the trouble is an œdematous swelling both within and without the prepuce, next colicky symptoms, the urine being discharged in drops or jerks, accompanied by groans. Palpation of the preputial cavity at this stage will reveal a painful and swollen state of the mucous membrane lining it, with the penis retracted as far as possible, the lining being covered with a black mass, while occasionally a more liquid purulent agent—an active competitor as regards odor to the decaying tooth—is encountered. Should the animal permit his penis to be drawn out, the swollen state of the glans, with its projecting, injected urethral opening, becomes apparent. Whenever this state, as just described, is allowed to go on without medical attention, ulceration and sloughing of the parts, and excessive granulations almost filling the preputial cavity, may follow.

On the whole but little trouble is experienced in effecting a cure. It is to be admitted that there is a tendency to chronicity, and in such cases where ulcerations are present in the preputial cavity decidedly active treatment becomes a leading feature. Balanitis and acrobustitis depending on new growths may resist all medication, amputation of the penis being a last resource.

Most cases will make a prompt recovery when thoroughly cleansed with warm soapsuds, adding about 5 per cent. soda carbonate, the latter addition helping to saponify the sebaceous matter. The whole cavity after having been thus cleansed is flushed with a 2 per cent. solution of germol or creolin, or, what has given me the best results, protargol 8 parts, water 92 parts. In those cases with a tendency toward infiltration, that is, whenever an active inflammation is present, slight vesication, followed with scarification of the sheath about four hours later, will give excellent results.

Excessive granulations have to be removed and the parts treated with absorbent and antiseptic applications, as irrigation with a 3 per cent. formalin solution or the above protargol solution, followed by some such powder as tannic acid 16 drachms, pulverized sugar 3 ounces, dusted upon the parts.

---

Dr. Fred. M. Hovey, of Marshall, Mich., reports a third outbreak of anthrax or braxy near that point. Microscopical examinations have been made, verifying the diagnosis.

